# AuDis: an automatic CRF-enhanced disease normalization in biomedical text

**DOI:** 10.1093/database/baw091

**Published:** 2016-06-07

**Authors:** Hsin-Chun Lee, Yi-Yu Hsu, Hung-Yu Kao

**Affiliations:** ^1^Institute of Medical Informatics, National Cheng Kung University, Tainan, Taiwan, R.O.C; ^2^Department of Computer Science and Information Engineering, National Cheng Kung University, Tainan, Taiwan, R.O.C

## Abstract

Diseases play central roles in many areas of biomedical research and healthcare. Consequently, aggregating the disease knowledge and treatment research reports becomes an extremely critical issue, especially in rapid-growth knowledge bases (e.g. PubMed). We therefore developed a system, AuDis, for disease mention recognition and normalization in biomedical texts. Our system utilizes an order two conditional random fields model. To optimize the results, we customize several post-processing steps, including abbreviation resolution, consistency improvement and stopwords filtering. As the official evaluation on the CDR task in BioCreative V, AuDis obtained the best performance (86.46% of F-score) among 40 runs (16 unique teams) on disease normalization of the DNER sub task. These results suggest that AuDis is a high-performance recognition system for disease recognition and normalization from biomedical literature.

**Database URL:** http://ikmlab.csie.ncku.edu.tw/CDR2015/AuDis.html

## Introduction

**Figure 1.**
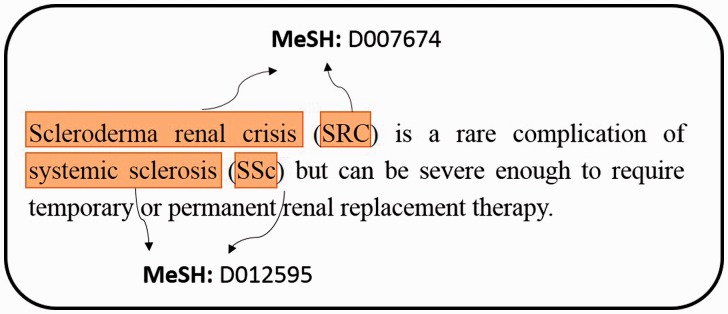
An example of extracting mentions from PubMed literature, and assigning MeSH concept identifier for each mention.

In the biomedical field, it has a rapid and exponential growth of producing large-scale biomedical literature (1). From the investigation of PubMed queries, the topics of chemical/drug, gene/protein and disease are within top 5 (2). Over the last decade, an online database named Comparative Toxicogenomics Database (CTD) (3) provided a cross-species resource for building interaction networks containing manually curated information, such as chemical–gene/protein interactions, chemical–disease and gene–disease relationships. With further curated information, the researchers could understand how environmental exposures affect human health. However, the manual curation of literature is usually time-consuming and low efficient because a rapid literature growth in the biomedical field causes problems on aggregating knowledge to biocurators. To facilitate the integration of biomedical articles, developing an automatic annotation system will effectively help biocurators to curate the relations between bioconcepts. For example, chemicals, diseases, and their relations play central roles in many areas of biomedical research and healthcare. Before extracting their relations from PubMed articles, the system has to retrieve the mentions of bioconcepts from unstructured free texts and assign the mention a relative database identifier (e.g. MeSH) as illustrated in Figure 1.

The process of biocuration is critical and complicated because it involves the problems of natural language processing, such as information retrieval, information extraction and speech processing. In past few years, the recognition and normalization of bioconcepts in biomedical literature have attracted attention, such as chemicals (e.g. tmChem, ChemSpot etc.) (4–6), diseases (e.g. DNorm) (7), genes (GNormPlus, GNAT etc.) (8–12), species (e.g. SR4GN, LINNAEUS etc.) (13–15), variations (e.g. tmVar, MutationFinder etc.) (16–18) and composites (e.g. SimConcept) (19), respectively. When biocurators investigate biomedical articles, disease mentions are important in many lines of inquiry involving diseases, including etiology (e.g. gene-variation-disease relationships) and clinical aspects (e.g. diagnosis, prevention and treatment) (20).

In the biomedical texts (e.g. literature and medical records), medical terminology should be collected and organized by the schemes of medical nomenclature. Medical terminology is a language utilized to describe human body (e.g. ‘cardio’ refers to ‘heart’ which was combined by ‘cor’ in Latin and ‘kardia’ in Greek), disease, symptom or specific terminologies (e.g. ‘Code Blue’ is a specific type of emergencies often used to refer to a cardiopulmonary arrest). These medical terms are different from the English words we use in daily life. Further, medical terminologies are combined by medical roots, suffixes and prefixes derived from ancient Greek or classical Latin. Therefore, the medical compound words will generate a great diversity of disease names. Moreover, the medical compound words are easily comprehended by biomedical experts, but they are difficult to be effectively recognized by machine learning.

To approach the biocuration of disease names, we defined four major variation challenges which disease mention recognition would face. (i) Disease terminology: disease names naturally exhibit a complicate and inconsistent terminology problem. For example, ‘cancer’, ‘carcinoma’ and ‘malignant tumor’ share a similar meaning (20). (ii) Combination word: as principles of disease word formation, they are mostly composed of prefix, suffix and root. For instance, the word ‘hyper-’ represents overactive, therefore, ‘hypertension’ means high blood pressure. For another example, ‘nephritis’ and ‘nephropathy’ are the root word ‘nephro’ combined with ‘-itis’ and ‘-pathy’, which mean inflammation and disease. Prefixes and Suffixes also have droppable –o- which always acts as a joint-stem to connect two consonantal roots (e.g. ‘Cardiology’ is combined by ‘cardi(o)’ and ‘logy’ (iii) Abbreviation: disease abbreviations are frequently used in text (e.g. ‘HD’ presents ’Huntington disease’) which may be ambiguous with other concepts (e.g. gene). (ii) Composite disease mention: a coordination ellipsis which refers to two or more diseases. For example, ‘ovarian and peritoneal cancer’ indicates that two individual diseases are MeSH: D010051 (Ovarian Neoplasms) and MeSH: D010534 (Peritoneal Neoplasms), respectively.

In recent years, the aforementioned problems can be solved by controlled vocabulary (e.g. MeSH) or on-hand tools (e.g. BIOADI) (21). The BIOADI corpus is used to detect abbreviations (Short Form, SF) and their original names (Long Form, LF) in each abstract. On the other hand, assigning a best-matching database identifier to each mention is a complicated issue because there are many ancestors and descendants in the database tree structure. For instance, the same concept name will belong to different categories. For example, the concept name ‘Kidney Diseases (MeSH: D007674)’ is under two categories ‘Male Urogenital Diseases’ and ‘Female Urogenital Diseases and Pregnancy Complications’, but they have the same descendants. In addition, mentions are difficult to exact match concept names, and assign their database identifiers even though the database contains synonyms of each concept name as well. From our observations, most problems are partial matching and overlapping. For example, ‘hemorrhagic bronchopneumonia’ has a describing word (or an adjective). In this case, the disease mention should belong to ‘bronchopneumonia (MeSH: D001996)’. Furthermore, ‘cognitive disorders’ has a part-of-speech variation problem, and the disease mention should belong to ‘Cognition Disorders (MeSH: D003072)’. In contrast, the different writing type between American and British English could also cause problems (e.g. ‘Hypokalaemia’ and ‘Hypokalemia’ (MeSH: D007008)), abbreviations (e.g. ‘LV’ should be returned to its long form ‘Leukocytoclastic Vasculitides’. Last but not least, the most difficult problem is the compound terms, such as ‘vestibulotoxicity (MeSH: D015837)’ are composed of ‘vestibul(ar)’, ‘oto’ and ‘toxicity’.

Continuing the previous BioCreative IV CTD task, the BioCreative V CDR Track focuses on the two topics: Disease Named Entity Recognition and Normalization (DNER) and Chemical-induced-disease relation (CID). Meanwhile, the disease named entity recognition is difficult and may affect the performance in downstream information extraction (e.g. relation extraction) according to the four variation challenges and the lack of a large-scale training corpus. In previous works, most of researches are devoted to optimize disease mentions extraction problem, but how to assign a suitable database identifier to each mention is a current and critical work which was not solved effectively before.

In our participation of DNER subtask in the CDR track, we not only developed a machine learning-based disease recognition system to deal with the four major variation challenges but also attempted to extend a dictionary-look up approach, which can provide a simple, high-speed and outstanding performance system to effectively solve the disease name extraction problems and inflexible dictionary-based approach problems in the normalization step.

In this work, we propose a system AuDis equipped with recognition and normalization methods. The brief processing steps of AuDis are as below: (i) select significant features related to diseases for CRFs models, (ii) take three post-processing steps after CRF tagging: consistency improvement, abbreviation resolution, stopwords filtering, (iii) perform a lexicon extension and a dictionary-lookup in our normalization step. As the official evaluation on the CDR task in BioCreative V, AuDis not only presented a better performance than DNorm (80.64% of F-score) and also obtained the best performance (86.46% of F-score) among 40 runs (16 unique teams) on the disease normalization of the DNER sub task.

### Related work

In previous works, there are many efforts on genes and chemicals but a fewer attempts on disease named entity recognition and normalization. Nevertheless, many disease terminology resources are available such as MeSH, UMLS, SNOMED-CT (22) and Disease Ontology (23). In recent years, CTD focuses on curating disease information, and released an unique resource ‘MEDIC’, a disease vocabulary, combined with the Online Mendelian Inheritance in Man (OMIM) and the ‘Diseases’ branch of the National Library of Medicine’s Medical Subject Headers (MeSH). Moreover, the NCBI provides a disease corpus consisting of 793 PubMed abstracts with 6,892 disease mentions (24), and it could be split into three subsets: 593 articles for a training set, and 100 articles for a development set and 100 articles for a test set. The corpus is fully annotated at the mention and concept level to serve as a research resource for the biomedical natural language processing community.

DNorm (7) is a state-of-the-art disease normalization tool, which was released from NCBI. It is the first machine learning approach which overturns the typical method such as a dictionary-lookup, accounting the string similarity of query terms to the disease concept name and its recognized synonyms, and a semantic-based ranking algorithm. Based on pairwise learning to rank, DNorm presents a high-performing and mathematically principled framework for learning similarities between mentions and concept names directly from training data in normalization for disease names, and it is also a strong benchmark of DNER subtask on BioCreative V CDR Track.

In participation of the DNER sub task in CDR track, here we discuss some methods and the performance derived from other multiple teams. LeadMine (Team 304) (25) used a dictionary/grammar based approach collected from MeSH, Disease Ontology. They especially focused on the source derived from Wikipedia for recognition and normalization, and obtained F-score of 86.12%. Team 277 (26) used CRF models to recognize disease mentions. In addition to MeSH lexicons, they built the semantic extensions by adding corpus-derived semantic variants (from CDR and NCBI) to MeSH, such as automatic translation of medical root words and affixes to potential variants. Then they used similarity-based methods for normalization, and finally obtained F-score of 85.56%; Team 363 (27) used ExB‘s existing NER ensemble framework and combined the second-order (i.e. ExBCRF) Conditional Random Fields (CRFs) libraries using linguistic features and word dependency to extract mentions. Then they looked up mentions in a dictionary collected from the CDR corpus. Their system could detect common variations and specificity of each term, and obtained F-score of 85.38% in the official run.

The submissions for three runs are combined with three different training sets from our system. We not only collected multiple lexicons but also extracted some significant features for our CRF modules. After extracting disease names, we took three post-processing steps and used a dictionary-lookup approach in normalization step. One of our submissions achieved the highest F-score (86.46%).

## Methods

### DNER dataset

The datasets we used are released from the CDR corpus (28) which is developed by a group of experts and annotators from MeSH and Comparative Toxicogenomics Database (CTD). The CDR corpus (inter-annotator agreement (IAA) scores: 88.75%) consists of 1500 articles from PubMed with manual annotated entities of 4409 chemicals, 5818 diseases and 3116 chemical–disease interactions, and it is divided into three subsets: training, development and test sets with 500 articles, respectively. Note that 1400 articles (400 for test set) are selected from the existing curated data of CTD-Pfizer corpus. The additional 100 articles for the test set are generated during the CDR challenge, and made public after the challenge was completed. The detailed description of dataset is illustrated in Table 1 as below.

**Table 1.** Statistics of CDR corpus

Task Dataset	Articles	Chemical		Disease		CID
		Mention	ID	Mention	ID	Relation
Training	500	5203	1467	4182	1965	1038
Development	500	5347	1507	4244	1865	1012
Test	500	5385	1435	4424	1988	1066

### System description

**Figure 2.**
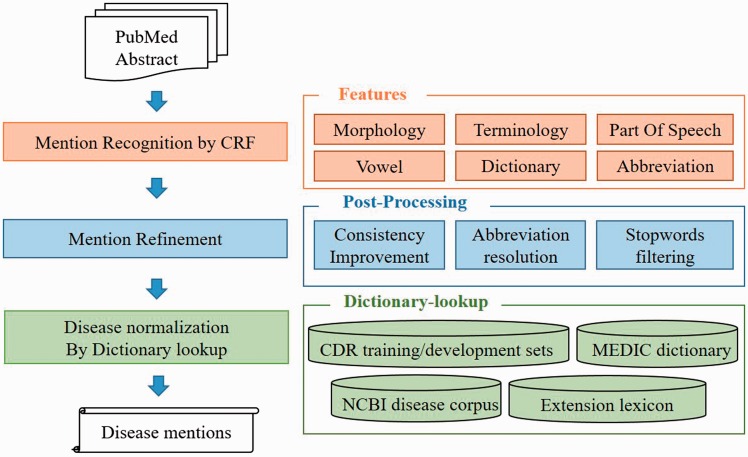
The overall architecture of the AuDis.

To handle the DNER task, we defined a semantic based recognition method which contains two individual modules (Figure 2). (i) Disease name recognition. We defined recognition model based on the linear-chain conditional random fields (CRFs) (29) with rich features and used two lexicons [NCBI Disease corpus (24), MEDIC (30)] to generate our CRFs dictionary features. Moreover, we also employed multiple post-processing steps, consistency improvement, abbreviation resolution, stopwords filtering, to further optimize the recognition results. (ii) Disease name normalization. To normalize disease mentions to specific concepts in existed repository, we developed a dictionary-lookup method based on the collection of MEDIC, NCBI disease corpus and CDR task released corpus (training set and development set) and our own extension dictionary. MEDIC is a well-known resource which uses a modified subset of descriptors from the ‘Diseases’ [C] branch of MeSH and OMIM identifiers to organize disease concepts, and we utilized the version released on 4 June 2015.

**Figure 3.**
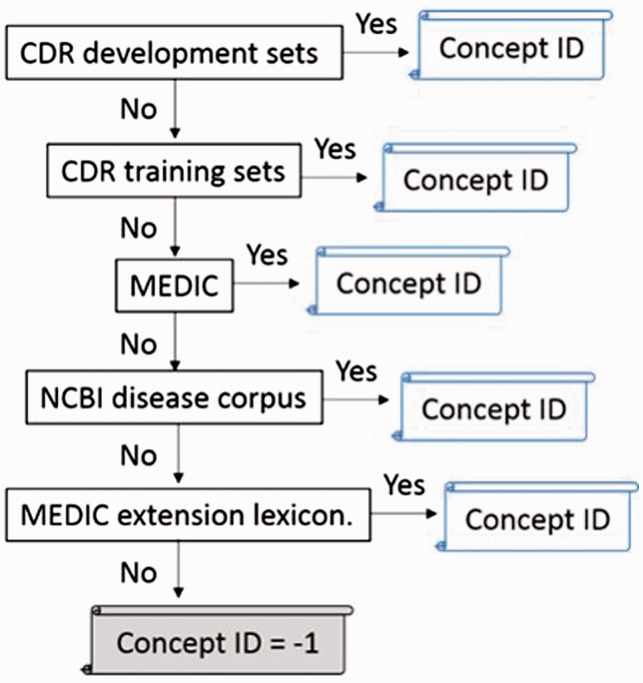
The flowchart of normalization step.

### Disease recognition: CRF module

A CRF is a statistical sequence-labeling algorithm which was introduced by Lafferty *et al.* (2001), and it has been applied in natural language processing and bioinformatics (e.g. entities extraction in biomedical literature). It is an arbitrary undirected probabilistic models for computing the conditional probability distribution $p\left(Y|X\right)$ of a random variables (label sequence) Y given the input X which is also called the observation sequence. The general model formulation of linear-chain CRFs is derived:

$$p\left(Y|X\right)=\;\frac{1}{Z(X)}\exp\left(f\left(Y,X\right)\right)=\frac{exp(f\left(Y,X\right))}{\sum_{Y'}exp(f\left(Y^{'},X\right))}$$

where $Y=\left\{y_{1},y_{2},\ldots ,y_{n}\right\}$ is a label sequence from an observation sequence $X=\left\{x_{1},x_{2},\ldots ,x_{n}\right\}$ which means a token sequence. $f\left(Y,X\right)=\sum_{j=1}^{n}\sum_{i=1}^{m}\omega_{i}f_{i}\left(y_{i},y_{i-1},X\right)$, a weighted feature function consists of state and transition feature function between position i and i-1 where each input $x_{i}$ is a vector of real-valued features known as containing all the components of the global observation sequence X, and a feature weight can learn from training data by a limited-memory Broyden–Fletcher–Goldfarb–Shanno (L-BFGS) (31). Finally, $Z\left(X\right)$ is the normalization function to [0, 1] given by $\sum_{Y'}\exp\left(\sum_{j=1}^{n}\sum_{i=1}^{m}\omega_{i}f_{i}\left(y_{i},y_{i-1},X\right)\right)$

In this model, we leveraged the CRF ++ toolkit (https://taku910.github.io/crfpp/) to train our disease named entity recognition model. The model also utilizes BIEO states (B: begin, I: insides, E: end and O: outside) and a second order template of CRFs, which assists our system to recognize a disease named entity. Note that we only use state ‘B’ (begin) as the single word of disease name.

### Pre-processing: tokenization

In the pre-processing stage, we break tokens at white spaces, punctuations and digits. Table 2 presents the concepts of breaking tokens. Then, the BIEO state of disease mention ‘hepatitis’, ‘autoimmune hemolytic anemia’ and ‘erythroblastocytopenia’ are tagged, as shown in Table 3. Each token will not only have its feature vector but also be tagged a BIEO state (label). The state ‘O’ represents the outside token which is not related to the disease mention. Note that the chemical mention would be tagged as an outside state, even though it is also a correct named-entity in the CDR corpus.

**Table 2.** The example of PMID: 9625142

Title	Acute hepatitis, autoimmune hemolytic anemia and erythroblastocytopenia induced by ceftriaxone.				

Abstract	An 80-yr-old man developed acute hepatitis shortly after ingesting oral ceftriaxone. Although the transaminases gradually returned to ……				

PMID	Start offset	End offset	Mention	Mention type	Database identifier
9625142	6	15	hepatitis	Disease	D056486
9625142	17	44	autoimmune hemolytic anemia	Disease	D000744
9625142	50	72	erythroblastocytopenia	Disease	−1
9625142	130	139	hepatitis	Disease	D056486

**Table 3.** The example of breaking the title of PMID: 9625142 into tokens, and tagging label to each token

Acute	Hepatitis	,	Autoimmune	Hemolytic	Anemia	,
O	B	O	B	I	E	O
and	erythroblastocytopenia	induced	by	ceftriaxone	.	
**O**	**B**	**O**	**O**	**O**	**O**

**Table 4.** The groups of three disease types

Groups	Conditions
Disease terminologies	Impairment, nausea, vomiting, disease, cancer, toxicity, insufficiency, effusion, deficit, dysfunction, injury, pain, neurotoxicity, infect, syndrome, symptom, hyperplasia, retinoblastoma, defect, disorder, failure, hamartoma, hepatitis, tumor, damage, illness, abnormality, tumour, abortion
Body part	Pulmonary, neuronocular, orbital, breast, renal, hepatic, liver, hart, eye, pulmonary, ureter, bladder, pleural, pericardial, colorectal, head, neck, pancreaticobiliary, cardiac, leg, back, cardiovascular, gastrointestinal, myocardial, kidney, bile, intrahepatic, extrahepatic, memorygastric
Human ability	Visual, auditory, learning, opisthotonu, sensory, motor, memory, social, emotion

### Feature group

The mention recognition issue has been addressed for many years. To reduce the development cost, we adapted the feature extraction from three recent recognition tools [i.e. CoINNER (32), tmChem (4), tmVar (16)]. In addition, we specifically expand our CRFs features to be suitable for assisting disease name extraction. The six significant feature groups we utilized in this model are described as below:

1. *Morphology*: We employed the general features including the original tokens, stemmed tokens (extracted by Snowball library) and its prefixes/suffixes (length 1–5).
2. *Terminology*: We manually gathered some common conditions from CTD, and defined three significant types, disease terminologies, body part and human ability to determine whether each token matches the condition we set. The detailed conditions that manually gathered from CTD are described in Table 4.
3. *Part* *of* *speech*: A series of binary features for each part of speech.
4. *Vowel*: We defined a frame to represent the token. Continued vowels change to only one ‘-’. For example, the words ‘tumor’ and ‘tumour’ are turned into the same frame ‘t-m-r’. Taking this definition, we could modify the divergence between American and British English.
5. *Dictionary-lookup*: We used the CTD disease vocabulary (MEDIC) and NCBI disease corpus as features. Furthermore, we set the length parameter which is > 3 to avoid false positives (i.e. an abbreviation of other concepts).
6. *Abbreviation*: We also annotated abbreviations and full names which are detected by BIOADI (21). Note that we only annotated them when they were detected in that article. That is, to avoid tagger inconsistency, the abbreviations or full names (e.g. ‘congestive heart failure’) will not be annotated even if they were detected in other articles.

### Post-processing for disease recognition

We employed a post-processing to improve the recognition results. In the first step, we improved the consistency by tagging all instances of a disease mention if the proportion of each mention is at least α within an article. As shown in Table 2, a disease term ‘hepatitis’ appears two times in an article, but our CRF model only recognizes one of all mentions in title. Thus, $p\left(hepatitis\right)$ is$\;\left(1/2\right)=0.5$, and the missed instances would be added in the recognition results when the ‘hepatitis’ occupy 50% within an article over the threshold (α=0.25) we set. This step could assist AuDis to raise the performance of Recall and become a robust recognition system, even though it would not play an important role in the normalization step.

$$p\left(m\right)=\frac{Quantity\;of\;CRF\;tagging}{Actual\;quantity\;of\;each\;mention\;}>\alpha ,\;where\;\alpha =0.25$$

Next, we used an abbreviation detection tool – BIOADI (21) to deal with the abbreviation challenge. We defined three rules in recognizing the correct disease abbreviation pairs. First, the instance was not recognized by CRF models, but was detected by BIOADI. All pairs would be annotated as disease mention when the lexicon contains the long form. On the other hand, if the long form of the pair is recognized as a disease mention by CRF models, all instances of the abbreviation pairs including both long and short forms are recognized as diseases. Besides, when the short form is recognized as a disease mention, the long form would be also recognized as a disease mention if the long form contains a disease terminology, such as ‘disease’, ‘cancer’, ‘syndrome’, ‘symptom’, ‘tumor’, ‘deficiency’ and ‘disorder’.

We also made the overlap modifications in the abbreviation resolution step. For example, when tagging the short form ‘PD’ in PMID: 19234905, the mention ‘UPDRS’ should not be tagged even though the word ‘PD’ is in ‘UPDRS’. That is, we should avoid tagging the mention which contains the character of the alphabet or digit in the string. The final step of our post-processing is stopwords filtering which can be found at the MySQL website (https://dev.mysql.com/doc/refman/5.1/en/fulltext-stopwords.html).

### Disease normalization

To identify relevant MeSH identifiers for recognized disease mentions, we developed a dictionary-lookup approach. The disease name lexicons are collected from the MEDIC (Comparative Toxicogenomics Database), the NCBI disease corpus, and the CDR training/development sets (28) which are adapted from a subset of the BioCreative IV CTD training corpus. All the disease names and their synonyms are utilized for normalization. Note that all the punctuations and white spaces are removed.

Since the term variation is a very critical issue in the disease recognition, and it would further affect the performance (especially the Recall) of normalization. We therefore generalize two common synonym problems from our false negative results when the CDR development set is regarded as our test set. In our observations, ‘disease’ and ‘failure’ are highly variant as shown below: ‘Infection’, ‘damage’, ‘abnormalities’, ‘disorder’, ‘impairment’, ‘loss’, ‘complication’, ‘injury’, ‘deficit’, ‘anomaly’ and ‘symptom’ are regarded as the synonyms of ‘disease’; likewise, ‘deterioration’, ‘diminished’, ‘reduced’, ‘subnormal’, ‘dysfunction’, ‘degeneration’, ‘decrease’, ‘impairment’, ‘insufficiency’, ‘weakness’, ‘lesion’ are regarded as the synonym of ‘failure’. That is, we exhaustively gathered those synonyms, and extended the MEDIC lexicons more flexibly. All plurals of those synonyms are appended as well.

However, there are some ambiguous concepts among our lexicons. To address this problem, we defined different orders to approach the best performance of nomalization, as shown in Table 5. In addition, extracted mentions in texts and disease synonyms in lexicons are changed to lowercase. The disease identifier is assigned to the exact matched mention. The priorities of dictionary-lookup approach we used in the official evaluation step are shown below: CDR development sets > CDR training sets > MEDIC > NCBI disease corpus > MEDIC extension lexicon. Once a mention indicates to more than two or more identifiers, the identifier in a higher priority lexicon would be admitted.

**Table 5.** The performance of four different orders on the CDR development set

Run	Different order of four lexicons	Precision	Recall	F-score
1	Train > 791 > MEDIC	0.8827	0.7909	0.8343
2	Train > MEDIC > 791	0.8832	0.7909	0.8345
3	Train > MEDIC > 791 > Extend	0.8791	0.8070	**0.8415**
4	MEDIC > Train > 791 > Extend	0.8567	0.7920	0.8231

The highest value is shown in bold.

Besides, if an abbreviation is not in the lexicons, our system would assign the identifier which matched by the long form. Finally, we defined two heuristic mention modifications to handle the American and British English issue (e.g. turn ‘ae’ into ‘e’) and the suffix issue (e.g. turn ‘-mia’ into ‘-mic’). In this step, a few mentions can be matched exactly if the original mentions cannot be matched correctly. Otherwise, our system would assign ‘-1’ if the mentions cannot be matched in lexicons.

## Result

In the DNER subset, *precision* (the fraction of retrieved instances that are relevant), *recall* (the fraction of relevant instances that are retrieved) and *F-score* (also called F1 score or F-measure, the harmonic mean of precision and recall) are used as the official evaluation criteria to our results. For evaluation, the organizers only focus on the results of relative database identifiers (i.e. Normalization results) to each abstract. That is, it considers the relevant concept identifiers without overlap. The false positive $\left(fp\right)$ results mean that the total number of false concept identifiers is retrieved from each abstracts. The false negative $\left(fn\right)$ results are defined as the total number of correct concept identifiers (i.e. gold standards) which are not retrieved from each abstracts. The formulations of precision $\left(p\right)$, recall $\left(r\right)$ and F-score $\left(f\right)$ are derived, respectively.

$$p=\frac{tp}{tp+fp}\;r=\frac{tp}{tp+fn}\;f=2*\frac{p*r}{p+r}$$

The official evaluation is done by an online testing. All participants of the CDR task should prepare a RESTful API equipped with their own recognition method for organizers to an online test. The performance of AuDis was officially evaluated by the BioCreative V CDR corpus (18). In the official three runs, we made up three different training sets to our CRF model. The detailed combinations for three runs and their performance are presented in Table 6.

**Table 6.** The performance of disease normalization on the CDR Testing for three runs

Run	Training set for CRF	Precision	Recall	F-score
1	Train	0.8942	0.8244	0.8579
2	Train + Dev	0.8963	0.8350	**0.8646**
3	Train + Dev + 791	0.8832	0.8365	0.8592

The highest value is shown in bold.

We used 1000 abstracts (500 abstracts in the training set and 500 abstracts in the development set) with human annotated gold standard for training, and tested on 500 abstracts. The results presented in Table 7 show the official evaluation results from the BioCreative V CDR track. Our system presents a better performance than DNorm and also obtained the best result among all participation runs on the DNER task (21).

**Table 7.** The performance of disease normalization on the CDR testing set, the results are the best submissions of all participating teams and the best result of our submission is the current setting of AuDis

Team	TP	FP	FN	Precision	Recall	F-score
AuDis	1660	192	328	89.63%	83.5%	86.46%
304	1713	277	275	86.08%	86.17%	86.12%
277	1629	191	359	89.51%	81.94%	85.56%
363	1606	168	382	90.53%	80.78%	85.38%
310	1627	247	361	86.82%	81.84%	84.26%
Average of all teams	1487	418	501	78.99%	74.81%	76.03%
*Baseline and strong benchmark*						
Dictionary-lookup	1341	1799	647	42.71%	67.45%	52.30%
DNorm	1593	370	395	81.15%	80.13%	80.64%

## Discussion

### Unique disease mentions in CDR corpus

Due to a large number of disease mentions in the CDR corpus, we make some comparisons with our training set and test set in two different views. When we analyzed non-repetitive disease mentions, there are 2044 and 1253 non-repetitive disease mentions in the training set and the test set, respectively. Note that there are 662 unique disease mentions in the test set, which the disease mentions are not found in the training set but they appear in the test set. The performance of AuDis shows that it could recognize 335 mentions among them from our CRF module.

The other analysis of unique disease mentions is from non-repetitive concept. It contains a number of 1088 (include ‘-1’) and 646 non-repetitive concept in the training set and the test set, respectively. The test set also contains 180 unique disease mentions, and AuDis could extract a number of 115 unique disease mentions among them.

### Evaluation of feature group

To assess the importance of our features, we used a leave-one-out cross-validation to observe the best performance in our six features. The performance of each removed feature and their results are presented in Table 8. In this experiment, the dictionary-lookup feature plays a central role in our system and provides a solution to the lack of large-scale training corpus problem. Without the dictionary-lookup feature, the performance decreases around 6% of F-score and has a low recall, which means that a number of relative identifiers cannot be found.

**Table 8.** The performance of removing one of our six feature groups

Removed feature	Precision	Recall	F-score
All features	89.63%	83.5%	86.46%
Dictionary-lookup	91.67%	71.43%	80.29%
Morphology	88.23%	82.24%	85.13%
POS	88.93%	82.44%	85.57%
Vowel	89.09%	82.60%	85.72%
Abbreviation	89.05%	82.65%	85.73%
Terminology	89.15%	82.70%	85.80%

### Error analysis

**Figure 4.**
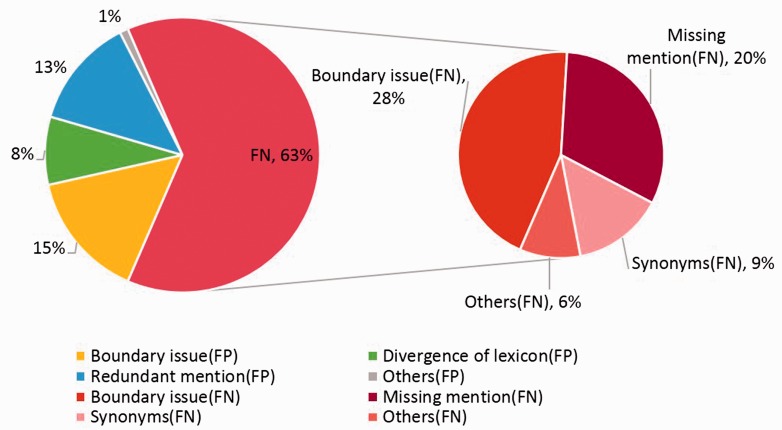
The eight categories of false positives and false negatives.

In the error analysis of AuDis, we focus on the results from our run2 (86.46% of F-score) and their false positives and false negatives on the CDR testing set. Note that disease mention extraction is also an important step because it may affect the performance in downstream information (i.e. Normalization and building relations between diseases and chemicals). Although we extracted some significant features to help our system recognize the correct mentions for normalization, we still have some problems, such as making numbers of partial match mentions and overlapping lexicons. Hence, we classify our results into several categories, such as boundary issues, divergence of lexicons, redundant mentions, missing mention, synonyms and others. In our observations, 63% are false negatives and 37% are false positives. The detailed description is shown in Figure 4.

The boundary issues are the major errors of our system, and the problem could be traced to the recognition of our CRFs module, which 15% errors are false positives and 28% are false negatives. That is, some mentions have more or less adjective words, such as ‘acute’ or ‘end-stage’. These mentions would increase false positives and false negatives at the same time. For instance, disease mention ‘encephalopathy’ in PMID: 17356399 was tagged a relative identifier ‘D001927’. However, the correct mention should be extracted as ‘Acute encephalopathy’ and should be assigned an identifier ‘D020803’, or ‘acute hepatitis (D017114)’ should be tagged as ‘hepatitis (D056486)’ correctly. This issue is complicated because we do not have a standard to extract mentions or not. On the other hand, some mentions should be further normalized, such as ‘sensory and motor dysfunction (D007049)’ or ‘central nervous system leukemia (D002493)’. Since these mentions have new sequences, our CRF modules cannot control the OFFSET exactly. As the examples we mentioned above, our system will only extracts mentions such as ‘motor dysfunction (D020258)’ and ‘leukemia (D007938)’ and result in false positive.

The missing mentions and redundant mentions are 20% in false negatives and 13% in false positives, respectively. When analyzing the missing mentions, they can also be classified into several categories, such as synonyms, abbreviations, morphological variations and the most difficult issue of the combinations of prefix, suffix and root. Because of the lack of the training corpus, some mentions have been ignored, such as ‘metabolic derangement (D008659)’ in PMID: 1756784, ‘FSGS (D005923)’ in PMID: 3323599, ‘gram-negative bacillary infections (D016905)’ in PMID: 256433. The combination word, such as ‘Blepharoconjunctivitis (D003231)’ in PMID: 2931989, is still difficult to determine its concept name because some disease mentions are generated arbitrarily according to the rules of medical nomenclature.

Using a dictionary-lookup approach in the normalization step, the system resulted in 8% divergence of concept identifiers because we used the multiple lexicons. Although we set the best priority to our dictionary-lookup approach, there are still noises. For example, some mentions, such as ‘renal disease’, are assigned to ‘D052177’ from the CDR development set, but the database identifier ‘D007674’ from the NCBI disease corpus is assigned correctly in most cases. The other example is the mention ‘parkinsonian (D010300)’, which has two different identifiers ‘D010300’ and ‘D020734’ from the CDR training set and development set, respectively. Thus, the divergence of lexicons becomes apparent, especially between the CDR training set, the development set, the CDR corpus and the NCBI corpus.

When analyzing the error cases of synonyms, we found that the synonym problems are different from the missing mentions. Here, our synonyms are defined as the mentions which are extracted correctly by our CRF modules but cannot be assigned a correct identifier from our normalization step. Furthermore, we observe that some mentions are similar to the concept names from MEDIC. Although we tried to extend our lexicons, some ambiguous concepts such as ‘abnormal ocular motility (D015835)’ in PMID: 12912689, ‘myocardial degeneration (D009202)’ and ‘renal disturbance (D007674)’ in PMID: 20859899 could not be solved.

As for the abbreviation problems, we found that most problems occur in the different disease full names. For example, ‘PD’ in PMID: 17445520 indicates that ‘panic disorder’, but our system assigned a relative database identifier ‘D010300’, which is an abbreviation of ‘Parkinson's Disorder’. We believe that the problems of abbreviation synonyms could be solved after updating our normalization rules. Moreover, punctuation problems arise in the CDR training set and the development set, such as ‘nausea, vomiting’. In our experience, the mentions combined with ‘nausea’ and ‘vomiting’ should be assigned to an identifier ‘D020250’ rather than ‘D009325’ and ‘D014839’, which are derived from ‘nausea’ and ‘vomiting’, respectively. However, in the CDR test set, we should consider ‘nausea, vomiting’ as two individual mentions. The other example is ‘ischemia-reperfusion injury’, which also should be considered as two separate mentions without hyphen.

## Conclusions

We developed a disease recognition/normalization tool with a state-of-the-art performance and an efficient processing speed. In terms of efficiency, it typically takes <50 s to run 500 PubMed abstracts through the system on dual quad-core windows server with 24 GB RAMs. Then, we setup a RESTful API for end-users to access the service. According to the observed performance, we believe that AuDis would be a useful resource for text mining down-stream research. We proposed a robust CRF-based recognition module to precisely recognize disease mentions, and employed a post-processing to improve the recognition results. The recognition performance is 85.64% of F-score. In the normalization step, we present a dictionary-lookup approach which obtains 86.46% of F-score. However, the recall of our method is relatively lower. In our future work, we will focus on raising the performance of normalization by a robust machine learning approach.
